# ACE and response to pulmonary rehabilitation in COPD: two observational studies

**DOI:** 10.1136/bmjresp-2016-000165

**Published:** 2017-03-08

**Authors:** Samantha S C Kon, Caroline J Jolley, Dinesh Shrikrishna, Hugh E Montgomery, James R A Skipworth, Zudin Puthucheary, John Moxham, Michael I Polkey, William D-C Man, Nicholas S Hopkinson

**Affiliations:** 1 NIHR Respiratory Biomedical Research Unit, Royal Brompton and Harefield NHS Foundation Trust and Imperial College, London, UK; 2 Department of Respiratory Medicine, King's College Hospital, London, UK; 3 Institute for Human Health and Performance University College, London, UK

**Keywords:** Pulmonary Rehabilitation, COPD Pathology

## Abstract

**Introduction:**

Skeletal muscle impairment is an important feature of chronic obstructive pulmonary disease (COPD). Renin–angiotensin system activity influences muscle phenotype, so we wished to investigate whether it affects the response to pulmonary rehabilitation.

**Methods:**

Two studies are described; in the first, the response of 168 COPD patients (mean forced expiratory volume in one second 51.9% predicted) to pulmonary rehabilitation was compared between different ACE insertion/deletion polymorphism genotypes. In a second, independent COPD cohort (n=373), baseline characteristics and response to pulmonary rehabilitation were compared between COPD patients who were or were not taking ACE inhibitors or angiotensin receptor antagonists (ARB).

**Results:**

In study 1, the incremental shuttle walk distance improved to a similar extent in all three genotypes; DD/ID/II (n=48/91/29) 69(67)m, 61 (76)m and 78 (78)m, respectively, (p>0.05). In study 2, fat free mass index was higher in those on ACE-I/ARB (n=130) than those who were not (n=243), 17.8 (16.0, 19.8) kg m^−2^ vs 16.5 (14.9, 18.4) kg/m^2^ (p<0.001). However change in fat free mass, walking distance or quality of life in response to pulmonary rehabilitation did not differ between groups.

**Conclusions:**

While these data support a positive association of ACE-I/ARB treatment and body composition in COPD, neither treatment to reduce ACE activity nor ACE (I/D) genotype influence response to pulmonary rehabilitation.

**Key Messages**

- Treatment with an ACE-I/angiotensin receptor blocker is associated with higher fat free mass in people with COPD.
- Neither treatment with ACE-I/angiotensin receptor blocker nor ACE (I/D) polymorphism appear to influence response to pulmonary rehabilitation.

## Background

Skeletal muscle impairment is a common and important feature of chronic obstructive pulmonary disease (COPD), occurring in about one-third of patients irrespective of the severity of their airflow obstruction.^1–3^ It is associated with reduced exercise capacity,^4^ as well as impaired quality of life,^5^ and quadriceps weakness has been shown to predict mortality in COPD independent of lung function.^6^ Physical inactivity is clearly a key driver of muscle weakness in COPD,^3^ ^7^ ^8^ although other factors, for example inflammation, hypoxia and hormonal factors, as well as genetic predisposition, may be important.^5^ ^9–11^ Moreover, there is strong evidence that pulmonary rehabilitation (PR), a programme of supervised exercise and education, can produce significant improvements in quality of life, muscle strength and endurance, as well as exercise capacity, in patients with COPD.^12^ ^13^

The circulating (endocrine) renin–angiotensin system (RAS) plays an important role in circulatory homeostasis, degrading vasodilator bradykinin and synthesising vasoconstrictor (and renal sodium-retaining) angiotensin II. However, local RAS also exists in diverse tissues^14^ including skeletal muscle.^15^ The presence (insertion, I) rather than the absence (deletion, D) of a 287 base pair sequence in intron 16 of the human ACE inhibitors gene is associated with lower tissue^16^ ^17^ and circulating ACE activity.^18^ ^19^ In turn, an extensive literature supports an association between ACE genotype and physical performance, the I allele being associated with endurance performance, and the D with power/sprint performance.^19^ ^20^ The ACE (I/D) polymorphism, as well as polymorphisms of genes for bradykinin type 2 (BK(2)R) and vitamin D receptors, have been shown to influence strength and body composition in COPD.^9–11^ Furthermore, ACE-I use in patients with hypertension has been associated with preservation of quadriceps strength and walking speed compared to those on other medications or controls.^21^

Given these data, we wished to establish whether factors known to affect RAS activity might influence response to PR in patients with COPD. We investigated this by conducting two studies. The first explored the impact of the ACE (I/D) polymorphism on responses to PR in patients with COPD. The second, in a separate cohort, investigated the effects of concomitant ACE-I or angiotensin II receptor blocker (ARB) use in patients with COPD, testing the hypothesis that this would be associated with preserved fat free mass (FFM) and an enhanced response to PR.

## Methods

Study 1, investigating the effect of ACE genotype, was approved by the Ethics Committee of King's College Hospital (05/Q0703/134) and funded by The British Lung Foundation and started in 2004. Participants provided written informed consent. The study was retrospective and involved contacting patients with a clinical diagnosis of COPD who had completed a PR programme at King's College or Royal Brompton and Harefield Hospitals. The PR programmes consisted of an 8 week course of aerobic and strength activities with two supervised and one or more home sessions per week. The initial exercise prescription was based on the outcome of their baseline incremental shuttle walk test distance (ISWD),^22^ and workloads were increased through the programme as tolerated. Programmes were multidisciplinary with an educational component covering issues including exercise, medication use, diet and coping strategies. Patients with COPD who attended at least 75% of their scheduled rehabilitation sessions and had ISWD measured pre-rehabilitation and immediately post-rehabilitation were invited to take part. Either a blood sample or a mouth swab was obtained from each patient to collect cells from which the PCR was used to determine ACE genotype.^11^ None of the patients or clinical research staff involved in the study knew the genotype of participants until after the phenotypic outcomes database had been finalised.

The association of ACE genotype with baseline characteristics and response to PR was assessed across all three genotypes by ANOVA and also between those with or without the D or I allele by unpaired *t*-test. The primary end point was change in ISWD immediately after PR. A p value of <0.05 was taken as significant and StatView 4.0 used for analysis.

In study 2, routinely collected data from a different cohort of patients with COPD who had been referred for a course of PR at Harefield Hospital between 2009 and 2011 were used. The Ethics Committee of Royal Brompton Hospital has determined that ethical approval is not required for the retrospective analysis of routinely collected clinical data. The primary outcome was differences in the response to PR of ISWD between patients who were or were not on an ACE-I or ARB (determined by patient self-report). Additional outcomes were fat free mass index (FFMI) determined by bioelectrical impedance analysis using a disease-specific regression equation,^23^ and the chronic respiratory disease questionnaire (CRQ)^24^ and the COPD assessment test score (CAT).^13^ ^25^ Data from 72 of the patients in study 2 were included in a previous publication.^13^

## Results

### Study 1: effect of ACE genotype on response to PR

**Figure 1:**
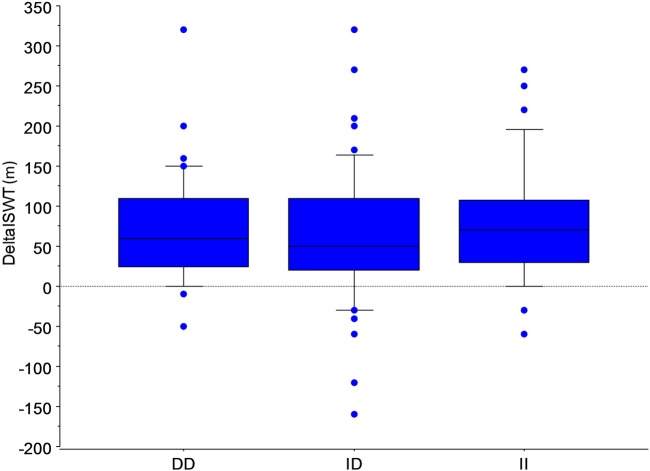
Plot of change in incremental shuttle walk test following pulmonary rehabilitation according to ACE (insertion/deletion) polymorphism. A total of 168 COPD patients took part, DD 48 (28%); ID 91 (53%); II 29 (19%). The horizontal line represents median value. Boxes represent 25th/75th centiles; whiskers 10th/90th centiles (ANOVA, p=0.5).

Data were available for 168 individuals who had participated in PR; 92 (53.8%) women, forced expiratory volume in one second (FEV_1_) 51.9 (22.7)% predicted. ACE genotypes were DD 48 (28%); ID 91 (53%); II 29 (19%) (table 1). There was no significant difference in patients' characteristics by ACE genotype either across all three possible genotypes (figure 1) or comparing those with or without an I or a D allele. Exercise capacity improved following PR in the whole study population with a mean increase in ISWD of 67.5(74.7)m and for each genotype (all p<0.0001), but the response did not differ significantly between genotypes (ANOVA, p=0.5).

**Table 1:** Study 1: patient characteristics for whole group and separated by ACE genotype

	All n=168	DD n=48 (28%)	ID n=91 (53%)	II n=29 (19%)
Age	68.7 (9.0)	69.2 (10.4)	67.8 (8.8)	71.0 (6.9)
Gender (n(%) female)	92 (54.8)	23 (47.9)	52 (57.1)	15 (51.7)
FEV_1_ (% predicted)	51.9 (22.7)	50.4 (22.3)	52.8 (24.4)	50.1 (18.7)
FEV_1_/FVC (%)	48.1 (17.6)	48.0 (17.7)	48.7 (19.3)	46.2 (13.2)
ISWD baseline (m)	251.4 (149.8)	261.7 (184.0)	253.0 (140.2)	223.4 (103.9)
ISWD end (m)	318.9 (170.6)	331.0 (200.0)	313.6 (165.3)	301.7 (109.4)
ΔISWD (m)	67.5 (74.7)	69.4 (66.6)	60.6 (76.2)	78.3 (78.2)
ΔISWD (%)	43.3 (66.7)	45.6 (76.9)	36.0 (53.2)	59.2 (84.5)

Values are mean (SD). All p>0.05 ANOVA across genotypes.

FEV_1_, forced expiratory volume in one second; FVC, forced vital capacity; ISWD, incremental shuttle walk test distance.

### Study 2: effect of ACE-I or ARB on response to PR

Baseline data from 373 consecutive COPD patients (213M:160F; mean age 68.3; median FEV_1_ 41% predicted) referred to an outpatient PR programme were analysed (table 2). Of these, 130 reported taking either an ACE-I (n=82), ARB (n=45) or both (n=3). The groups had similar gender distribution and long-term oral corticosteroid use. Patients on ACE-I or ARB were older, had less severe airflow obstruction but similar values for ISWD, CRQ, Medical Research Council dyspnoea score (MRC) and CAT. However, the patients receiving ACE-I or ARB had significantly higher FFM and FFMI 17.8 kg/m^2^ (16.0, 19.8) versus 16.5 kg/m^2^ (14.9, 18.4) (p<0.0001 when adjusted for difference in FEV_1_ and age).

**Table 2:** Study 2: baseline patient characteristics separated by whether patients were or were not taking an ACE-I or ARB.

	ARB or ACE-I n=130	No ARB or ACE-I n=243	p Value
Age (years)	71 (64, 78)	67.6 (9.8)	0.004
FEV_1_ (% predicted)	44.5 (32.3, 60.8)	39.0 (26.0, 58.5)	0.007
FFM (kg)	51.1 (11.2)	45.5 (40.1, 52.0)	<0.001
FFMI (kg/m^2^)	17.8 (16.0, 19.8)	16.5 (14.9, 18.4)	<0.001
ISWD (m)	140 (60, 250)	160 (80, 280)	0.10
CRQ	71.5 (55.8, 91.0)	68.0 (56.0, 87.0)	0.45
MRC dyspnoea score	4 (3, 5)	4 (3, 5)	0.79
CAT score	23.0 (8.0)	22.0 (7.0)	0.76

p Values are for unpaired *t*-tests. Data are presented as median (25th, 75th centiles) or (SD).

ACE-I, ACE inhibitor; ARB, AT II receptor antagonist; CAT, COPD assessment test score; CRQ, chronic respiratory disease questionnaire; FEV_1_, forced expiratory volume in one second; FVC, forced vital capacity; FFM, fat free mass; FFMI, fat free mass index; ISWD, incremental shuttle walk test distance.

A total of 255 patients completed the PR programme, 76 (30%) of whom were taking an ACE-I/ARB. Responses did not differ between those who were or were not on an ACE-I or ARB (table 3), with improvements in ISWD and CRQ exceeding the minimum clinically important difference.

**Table 3:** Study 2: response to pulmonary rehabilitation in COPD patients who were or were not taking an ACE-I or ARB.

	ARB or ACE-I n=76	No ARB or ACE-I n=179	p value
ΔFFM (kg)	−1.7 (2.7)	1.8 (1.4)	0.21
ΔISWD (m)	104 (21)	63 (15)	0.13
ΔCRQ	16.2 (3.8)	17.7 (2.5)	0.75

ACE-I, ACE inhibitors; ARB, AT II receptor antagonist; FFM, fat free mass; ISWD, incremental shuttle walk test distance; CRQ, chronic respiratory disease questionnaire.

All p>0.05. Data are presented as mean (SD).

## Discussion

The present data suggest that the beneficial response to PR in COPD patients was not strongly influenced by their ACE (I/D) genotype, or by pharmacological RAS antagonism. However, long-term use of an ACE-I/ARB was associated with relatively preserved FFM in patients referred for PR.

### Rationale for studying the RAS in COPD

In patients with COPD, the quadriceps muscle displays muscle fibre atrophy and a shift away from an endurance phenotype, with a reduced proportion of type I slow twitch, fatigue-resistant fibres together with reduced capillarity and oxidative enzymes.^26–30^ The RAS and thus ACE inhibitors and functional ACE gene polymorphisms have the potential to influence these processes through a number of mechanisms. These include effects on muscle atrophy/hypertrophy signalling, fibre shift, systemic inflammation and remodelling.^31^ Angiotensin II opposes the action of the insulin-like growth factor (IGF-1) system activating the ubiquitin-proteasome proteolytic pathway via IGF-1 and via NF-kB,^32^ ^33^ and IGF-1 levels are reduced in the quadriceps of COPD patients in the stable state compared to healthy controls.^34^ Increases in exercise capacity and fibre size in COPD patients undergoing PR are associated with upregulation of IGF-1 and its splice variant mechano-growth factor (MGF).^35^

The I allele of the ACE gene polymorphism is associated with a higher proportion of type I fibres,^36^ and there is evidence that ACE inhibitors and AT II receptor antagonists interact with peroxisome proliferator-activated receptors (PPARs)^37^ ^38^ which are major regulators of cell metabolism mediating type II (anaerobic) to type I (aerobic) fibre shift and regulate mitochondrial activity as well as muscle oxidative status.^39–41^ This is potentially of particular relevance in COPD given that PPAR-delta protein content is decreased in the skeletal muscle of these patients.^42^ The DD genotype has also been associated with systemic inflammation in COPD.^43^ In stable COPD patients, the deletion allele (D) of the ACE gene polymorphism has been associated with increased quadriceps strength, in contrast to age-matched healthy controls where this relationship was not observed.^11^

### Effect of ACE inhibition in COPD

In the present cross-sectional study, patients taking an ACE-I/ARB did have relatively preserved FFM, though this was not associated with differences in exercise capacity. Patients had not been randomly allocated to treatment so these data need to be treated with caution, but they are consistent with a beneficial effect of reduced ACE activity on body composition.^44^

In keeping with this, in healthy older people the use of ACE-I as a treatment for hypertension is associated with relative preservation of lower limb muscle mass,^45^ and with a reduced rate of loss of knee extensor strength^21^ compared to patients using other antihypertensives or to those not treated for hypertension. The ACE-I perindopril has been shown to increase 6 min walk distance in older people.^46^ A small number of studies have investigated the effects of RAS inhibition in COPD patients.^47–50^ In one study captopril improved pulmonary haemodynamics during exercise in patients with the ID or II genotype^47^ though other studies have not found similar effects.^51^ ^52^ A double-blind, placebo-controlled study by Di Marco *et al* evaluated the effects of 4 weeks treatment with enalapril on exercise performance in 21 COPD patients finding that it increased peak work rate in the treatment group compared to placebo, an effect not significantly modified by ACE genotype.^53^ A randomised controlled trial of fosinopril in 80 patients with COPD selected for quadriceps weakness found no benefit,^49^ and enalapril did not enhance the effect of PR on improvements in exercise performance in COPD.^50^ Of note, these two studies excluded people with a clinical indication for an ACE-I who, by definition, are the subject of the present paper.

Epidemiological data suggest a survival benefit in patients with COPD who are on an ACE-I.^54^ ^55^ However, in the present study, treatment with an ACE-I was not associated with greater strength or exercise capacity. Interestingly, the patients on ACE-I/ARB had less severe airflow obstruction but similar health status and dyspnoea. It is therefore possible that comorbidities such as cardiac impairment were contributing to their overall symptom burden and exercise limitation which might have had an effect on response to PR.

We found no association of the ACE(I/D) genotype with response to PR. This contrasts with Gosker *et al* who found, in a study of 95 COPD patients undergoing PR, that the improvement in peak VO_2_ during cycle ergometry was significantly less in patients with the DD genotype.^56^ However in that study, those with an I allele had a lower exercise capacity initially so may have been more detrained. The difference could also be due to the test modalities employed in the two studies (walking vs cycling) or a regression to the mean effect.

### Critique of methods

Functional exercise capacity is an integrative end point subject to respiratory, cardiac, skeletal muscle and motivational limitation, so the absence of an apparent effect of ACE genotype or ACE-I on response does not preclude the possibility of some physiological impact which might have been more apparent with a more controlled exercise end point such as metabolic parameters at a particular workload. Since the RAS is active at a number of levels, it may be that impacts on muscle strength, muscle endurance and the systemic and pulmonary vascular system may have opposing effects which a walking test cannot separate.

This paper addresses the question of whether either the genotype or treatment with drugs that influence the ACE system has an effect, in clinical practice, on outcome measures accepted as clinically relevant in international guidelines for PR—health status and exercise capacity assessed using a walking test.^13^ There is of course ongoing debate about the different information conveyed by laboratory and field tests of exercise performance as well as walking versus cycling, but there is certainly no reason to ascribe greater clinical relevance to VO_2_ max, etc than to performance on a field walking test when considering daily physical activity or patient-relevant outcomes.

Patient recruitment for the genotyping study was retrospective, so it is conceivable that some survival or other bias was in operation. Genotype data were not available for the cohort in study 2, so it is not possible to comment on possible interactions between genotype and treatment with ACE-I/ARB. It is possible that disease processes for which RAS antagonists were prescribed were themselves associated with differences in body composition. All participants were taking part in clinical PR programmes and data were entered prospectively, but because they were clinical programmes the full range of possible phenotypes were not recorded as might have been the case in a prospective study, such as exacerbation frequency and multimorbidities, as well as more detailed lung function parameters or gas transfer.^57^

Patient treatments were based on self-report, so it is possible that an effect of ACE-I or ARB was underestimated because of poor compliance with medication.

## Conclusions

Although treatment with an ACE-I/ARB was associated with a higher FFM in patients with COPD, neither the ACE (I/D) polymorphism nor treatment with an ACE/ARB appear to influence response to PR despite previous data showing an association with quadriceps strength. Although trial data do not support a beneficial effect from the addition of an ACE-I in COPD patients who do not have a conventional clinical indication,^49^ ^50^ the present data do not suggest that there is any advantage to avoiding or stopping ACE-I in COPD patients in whom they are indicated.
